# GPS/BDS triple-frequency cycle slip detection and repair based on moving window global search method

**DOI:** 10.1038/s41598-024-57063-5

**Published:** 2024-03-19

**Authors:** Jianying Wang, Dewu Huang

**Affiliations:** 1 School of Engineering, Yunnan College of Business Management, Kunming, 650500 China; 2 City College, Kunming University of Science and Technology, Kunming, 650106 China

**Keywords:** Civil engineering, Computational science

## Abstract

Cycle slip detection and repair are crucial steps in achieving high accuracy in Global Navigation Satellite System (GNSS) data processing. The use of Global Positioning System (GPS) and BeiDou Navigation Satellite System (BDS) triple frequency observations allows for more accurate detection and repair of cycle slips compared to single or dual frequency. This study presents a moving window global search method by selecting three sets of combined coefficients to construct geometry-free (GF) models to minimize the influence of the ionosphere, using a moving window to update the standard deviation of cycle slip estimation, applying the "3 $\upsigma$" criterion to constrain the range, and utilizing a global search method to detect and repair triple-frequency cycle slips. Through five sets of 1 Hz GNSS data experiments, the results demonstrate the effectiveness of this method in determining the position and size of triple-frequency cycle slips while avoiding multi-value problems. The detection success rate for GPS ranges from 98.0 to 100.0%, while BDS ranges from 92.0 to 100.0%. On average, GPS achieves a detection rate of 99.2%, and BDS reaches 96.7%, which is 0.8% and 1.8% higher than the direct rounding method, respectively. Compared to existing methods, it is also effective for the vast majority of small cycle slips within 2 cycles.

## Introduction

GPS in the United States and BDS in China are currently the most advanced GNSS systems in the world. During the GNSS observation process, the carrier signal may be obstructed or jammed, resulting in an interruption of the phase observation. When the receiver regains the signal, the slip from the previous signal is known as a cycle slip^1^. The detection and repair of GNSS cycle slips is a crucial issue in high-precision positioning research, as it directly affects the reliability of positioning results^2,3^. Therefore, it is essential to accurately detect and repair cycle slips. With the rapid development of multi-frequency satellites, there are now more combined models of multi-frequency in GNSS positioning, providing greater opportunities for cycle slip detection and repair^4,5^. However, current research on cycle slip detection and repair primarily focuses on single-frequency, dual-frequency, and triple-frequency systems^6^, with limited discussion on five-frequency systems^7^.

Single frequency cycle slip detection and repair commonly use single difference (SD), double difference (DD), and triple difference (TD) methods^8,9^, as well as fitting methods^10,11^. The difference method is suitable for detecting large cycle slips and is not sensitive to small ones. The fitting method has strict requirements for the order of fitting and the amount of data, and is prone to overfitting. Statistical testing and robust testing methods^12,13^ are used to extract the size of cycle slips by directly rounding after residual processing using the least squares method. While these methods can detect large cycle slips, they may not be sensitive enough to detect small cycle slips, particularly when they are close to the residual size. Huang and Huo ^14,15^ employed wavelet analysis for cycle slip detection. Although this method can detect most cycle slips, the selection of wavelet base can be challenging since different wavelet base functions have varying detection effects. For dual-frequency cycle slip detection, Sunil B. Bisnath^16^ proposed using the inter-station DD or TD method, which requires at least two receivers and is not suitable for single-station positioning. Banville and Langley^17^ propose a ground-based (GB) cycle slip detection method and apply it to different ionospheric environments. However, this method requires a significant amount of external information, such as precise orbit parameters and clock deviation parameters. As a result, it is more suitable for post-processing than real-time processing.Zhao et al.^18^ propose a three-step detection method for ultra-wide, wide, and narrow lanes. The method relies on ionospheric prediction, which is suitable for environments with low ionospheric activity or sampling rates but is not suitable for single-point precision positioning (PPP). Currently, the TurboEdit method^19–23^ is a widely used dual-frequency cycle slip detection and repair method that uses Melbourne-Wübbena (MW) and Geometry-Free (GF) models. Although this method is widely used in the practical world of engineering, it is not without its own problems. For instance, the dual-frequency combination wavelength is still relatively short, and at times, the pseudo-range error can significantly affect the detection outcome. At the same time, it is insensitive to cycle slips of the same size or special combination cycle slips on the dual-frequency, and it is prone to missed or false detections. Recently, scholars^24^ have made new attempts to detect cycle slips through graphical structural constraints. With the development of navigation systems, many studies have been conducted on the combination of GPS and BDS multi-frequency^25^. Currently, the combination coefficient method is widely used to detect triple frequency cycle slips. The selection of combination coefficients must ensure the integer characteristics of cycle slips, such as long wavelengths, low noise impact, and minimal influence from the ionosphere. Dai et al.^26^ propose a dual GF phase model for detecting and repairing triple-frequency cycle slips. The model utilises the least squares method to remove ambiguity correlation and determine the optimal value of cycle slips. While the model can detect most cycle slips, there are still a few insensitive ones that cannot be detected or repaired. Some scholars^27–32^ use a combination of pseudo-range-phase and GF models to detect triple-frequency cycle slips in BDS. The optimal combination coefficient is selected and possible cycle slips are searched for. Although most cycle slips can be detected, small cycle slips may be difficult to detect due to observation noise or special combination cycle slips.

This article presents a novel moving window global search algorithm for detecting and repairing GPS/BDS triple-frequency cycle slips. The algorithm utilizes three sets of frequency coefficient combinations, implements a moving window approach, applies integer constraints to cycle slip estimation, updates its range, and employs the global search method to simultaneously detect and repair triple-frequency cycle slips. Compared to other methods, this approach offers a simpler model and superior detection capabilities. One key advantage of this method is its ability to eliminate the need to consider the integer characteristics of combined cycle slips. It also effectively separates small cycle slips from observation noise, accurately constrains the real-time standard deviation of cycle slip sequences, and determines the position and size of each frequency's cycle slip without external conditions. Furthermore, it avoids the issue of multiple values for cycle slips.

## Method

### Cycle slip valuation

Given the triple-frequency ${f}_{i}\left(i=\mathrm{1,2},3\right)$ of GNSS, the equations for pseudo-range and phase observations can be expressed as follows:

1 $${P}_{i}=\rho +{\eta }_{i}{I}_{1}+{\varepsilon }_{{P}_{i}}$$

2 $${\lambda }_{i}{\varphi }_{i}=\rho -{\eta }_{i}{I}_{1}+{\lambda }_{i}{N}_{i}+{\lambda }_{i}{\varepsilon }_{{\varphi }_{i}}$$

where, ${P}_{i}$ represents pseudo-range observation value, ${\lambda }_{i}$ represents carrier wavelength, ${\varphi }_{i}$ represents the carrier phase observation, $\rho$ represents the geometric range, ${\varepsilon }_{{P}_{i}}$ and ${\varepsilon }_{{\varphi }_{i}}$ represent the observation noise of pseudo-range and phase respectively, ${N}_{i}$ is the integer ambiguity, ${\eta }_{i}={f}_{1}^{2}/{f}_{i}^{2}$, ${I}_{1}$ is the first-order ionospheric term parameters of ${f}_{1}$.

By combining Eqs. (1) and (2) and ignoring the influence of the ionosphere, we can calculate the difference between adjacent epochs ${t}_{1}$ and ${t}_{2}$. This will allow us to obtain the estimated cycle slip at epoch ${t}_{2}$:

3 $${\Delta N}_{i }\approx \Delta {\varphi }_{i}-\frac{\Delta {P}_{i}}{{\lambda }_{i}}$$

where, $${\Delta N}_{i }={N}_{i }\left({t}_{2}\right)-{N}_{i }\left({t}_{1}\right)$$, $${\Delta \varphi }_{i }={\varphi }_{i }\left({t}_{2}\right)-{\varphi }_{i }\left({t}_{1}\right)$$, $${\Delta P}_{i }={P}_{i }\left({t}_{2}\right)-{P}_{i }\left({t}_{1}\right)$$.

If the receiver receives pseudo-range ${P}_{i}$ on ${f}_{i}\left(i=\mathrm{1,2},3\right)$, it can take the average of the received multi-frequency pseudo-range observations instead of their respective ${P}_{i}$. If the receive can receive the GNSS P-code, replace the pseudo-range with the P-code. The triple-frequency cycle slip estimation represented by Eq. (3) can be expressed as:

4 $$\left\{\begin{array}{c}\Delta P=\frac{1}{3}\sum_{i=1}^{i=3}{P}_{i}\\ {\Delta N}_{i }\approx \Delta {\varphi }_{i}-\Delta P/{\lambda }_{i}\end{array}\right.$$

### Combined GF model

Linear combinations are employed in multi-frequency GNSS observations to mitigate the impact of certain parameters. The combination can be represented based on the triple-frequency phase observations:

5 $${\varnothing }_{\alpha }=\sum_{i=1}^{i=3}{\alpha }_{i}{\lambda }_{i}{\varphi }_{i}$$

where ${\upalpha }$ represents the combination coefficient. Expand the above Eq. (5) as:

6 $$\left\{\begin{array}{l}{\varnothing }_{\alpha }={\alpha }_{1}{\lambda }_{1}{\varphi }_{1}+{\alpha }_{2}{\lambda }_{2}{\varphi }_{2}+{\alpha }_{3}{\lambda }_{3}{\varphi }_{3}\\ {\varnothing }_{\alpha }=({\alpha }_{1}+{\alpha }_{2}+{\alpha }_{3})\rho +({\alpha }_{1}{\lambda }_{1}{N}_{1}+{\alpha }_{2}{\lambda }_{2}{N}_{2}+{\alpha }_{3}{\lambda }_{3}{N}_{3})-({\alpha }_{1}{\eta }_{1}+{\alpha }_{2}{\eta }_{2}+{\alpha }_{3}{\eta }_{3}){I}_{1}\end{array}\right.$$

If ${\alpha }_{1}+{\alpha }_{2}+{\alpha }_{3}=0$, the influence of geometric distance $\rho$ can be eliminated, and the difference between adjacent epochs can be obtained as:

7 $$\left\{\begin{array}{l}{\Delta \varnothing }_{\alpha }={\alpha }_{1}{\lambda }_{1}{\Delta \varphi }_{1}+{\alpha }_{2}{\lambda }_{2}\Delta {\varphi }_{2}+{\alpha }_{3}{\lambda }_{3}\Delta {\varphi }_{3}\\ {\Delta \varnothing }_{\alpha }=({\alpha }_{1}{\lambda }_{1}{\Delta N}_{1}+{\alpha }_{2}{\lambda }_{2}{\Delta N}_{2}+{\alpha }_{3}{\lambda }_{3}{\Delta N}_{3})-({\alpha }_{1}{\eta }_{1}+{\alpha }_{2}{\eta }_{2}+{\alpha }_{3}{\eta }_{3})\Delta {I}_{1}\end{array}\right.$$

If $({\lambda }_{1}{\eta }_{1}+{\lambda }_{2}{\eta }_{2}+{\lambda }_{3}{\eta }_{3})\Delta {I}_{1}\approx 0$, the influence of ionospheric residuals can be ignored. Combining the integer feature of cycle slips, the cycle slip estimation in Eq. (4) can be used to calculate the integer solution that satisfies Eq. (7), it is the cycle slip value.

### ${\varvec{\upalpha}}$ coefficient optimization

In order to eliminate the influence of geometric distance and weaken the influence of ionospheric residuals, multiple sets of coefficients ${\upalpha }$ can be found within a certain range, meeting the following conditions:

8 $$\left\{\begin{array}{c}{\alpha }_{1}+{\alpha }_{2}+{\alpha }_{3}=0\\ \left|{\alpha }_{1}{\eta }_{1}+{\alpha }_{2}{\eta }_{2}+{\alpha }_{3}{\eta }_{3}\right|=min\end{array}\right.$$

To prevent error amplification, select ${\upalpha }\in \left[-10 10\right]$ as the range for combination, and through comparison, obtain the relevant information of the combination coefficients of GPS and BDS triple-frequency phase observations, as shown in Tables 1 and 2. The table shows that the combination coefficient can eliminate the distance term and reduce the influence of the ionosphere by up to 80% compared to its original effect $\Delta {I}_{1}$. Based on the selected coefficients, cycle slip detection Eqs. (9) and (10) can be established, where $j$ in $\Delta {\varnothing }_{\alpha }^{j}$ represents the combination coefficient number.

**Table 1:** GPS combination coefficient.

$j$	${\alpha }_{1}$ ${f}_{1}=1575.42 {\text{MHz}}$	${\alpha }_{2}$ ${f}_{2}=1227.6 {\text{MHz}}$	${\alpha }_{3}$ ${f}_{3}=1176.45 {\text{MHz}}$	$\left|{\alpha }_{1}{\eta }_{1}+{\alpha }_{2}{\eta }_{2}+{\alpha }_{3}{\eta }_{3}\right|$	$|3{m}_{\Delta {\varnothing }_{\alpha }^{j}}|$
1	1	−6	5	0.084	0.084
2	1	−5	4	0.062	0.069
3	1	−4	3	0.208	0.054

**Table 2:** BDS combination coefficient.

$j$	${\alpha }_{1}$ ${f}_{1}=1561.098 {\text{MHz}}$	${\alpha }_{2}$ ${f}_{2}=1207.140 {\text{MHz}}$	${\alpha }_{3}$ ${f}_{3}=1268.52 {\text{MHz}}$	$\left|{\alpha }_{1}{\eta }_{1}+{\alpha }_{2}{\eta }_{2}+{\alpha }_{3}{\eta }_{3}\right|$	$|3{m}_{\Delta {\varnothing }_{\alpha }^{j}}|$
1	1	2	−3	0.199	0.039
2	1	3	−4	0.041	0.051
3	1	4	−5	0.117	0.066

9 $${\left[\begin{array}{l}\Delta {\varnothing }_{\alpha }^{1}\\ \Delta {\varnothing }_{\alpha }^{2}\\ \Delta {\varnothing }_{\alpha }^{3}\end{array}\right]}_{GPS}=\left[\begin{array}{c}1{\lambda }_{1}{\Delta \varphi }_{1}-6{\lambda }_{2}{\Delta \varphi }_{2}+5{\lambda }_{3}{\Delta \varphi }_{3}\\ 1{\lambda }_{1}{\Delta \varphi }_{1}-5{{\lambda }_{2}\Delta \varphi }_{2}+4{\lambda }_{3}{\Delta \varphi }_{3}\\ 1{\lambda }_{1}{\Delta \varphi }_{1}-4{\lambda }_{2}{\Delta \varphi }_{2}+3{{\lambda }_{3}\Delta \varphi }_{3}\end{array}\right]\approx \left[\begin{array}{ccc}{\lambda }_{1}& -6{\lambda }_{2}& 5{\lambda }_{3}\\ {\lambda }_{1}& -5{\lambda }_{2}& 4{\lambda }_{3}\\ {\lambda }_{1}& -4{\lambda }_{2}& 3{\lambda }_{3}\end{array}\right]{\left[\begin{array}{c}\Delta {N}_{1}\\ \Delta {N}_{2}\\ {\Delta N}_{3}\end{array}\right]}_{GPS}$$

10 $${\left[\begin{array}{l}\Delta {\varnothing }_{\alpha }^{1}\\ \Delta {\varnothing }_{\alpha }^{2}\\ \Delta {\varnothing }_{\alpha }^{3}\end{array}\right]}_{BDS}=\left[\begin{array}{c}1{\lambda }_{1}{\Delta \varphi }_{1}+2{\lambda }_{2}{\Delta \varphi }_{2}-3{\lambda }_{3}{\Delta \varphi }_{3}\\ 1{\lambda }_{1}{\Delta \varphi }_{1}+3{\lambda }_{2}{\Delta \varphi }_{2}-4{\lambda }_{3}{\Delta \varphi }_{3}\\ 1{\lambda }_{1}{\Delta \varphi }_{1}+4{\lambda }_{2}{\Delta \varphi }_{2}-5{{\lambda }_{3}\Delta \varphi }_{3}\end{array}\right]\approx \left[\begin{array}{ccc}{\lambda }_{1}& 2{\lambda }_{2}& -3{\lambda }_{3}\\ {\lambda }_{1}& 3{\lambda }_{2}& -4{\lambda }_{3}\\ {\lambda }_{1}& 4{\lambda }_{2}& -5{\lambda }_{3}\end{array}\right]{\left[\begin{array}{c}\Delta {N}_{1}\\ \Delta {N}_{2}\\ {\Delta N}_{3}\end{array}\right]}_{BDS}$$

### Moving window global search method

The cycle slip will cause an outlier in ${\Delta \varnothing }_{\alpha }$, and the location of the outlier is the epoch of the suspected cycle slip. By constraining the value range of ${\Delta N}_{i}$, the integer solution satisfying Eqs. (9) and (10) can be calculated to obtain the cycle slip value of GPS and BDS. Therefore, it is important to determine the location of the cycle slip in the sequence ${\Delta \varnothing }_{\alpha }$ and the value range of ${\Delta N}_{i}$.

**Figure 1:**
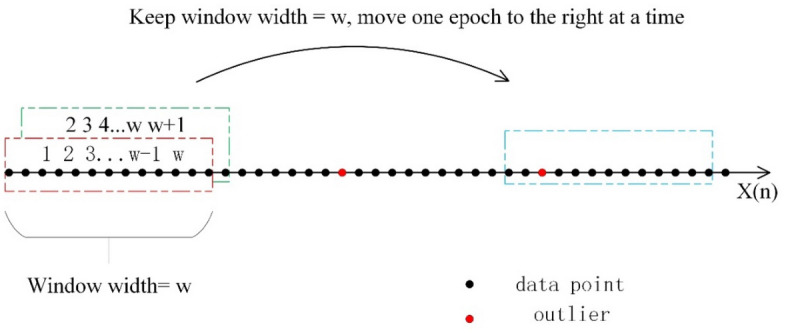
Schematic diagram of moving window.

Figure 1 illustrates the process of identifying outliers in a sequence X(n) of length n. The first step is to set a window of width w, starting from the first element of the sequence. This window consists of the first w elements of the sequence and is used to determine if the w + 1 element is an outlier. The window is then moved one element back, and the next w elements (2 ~ w + 1) are used to determine if the w + 2 element is an outlier, and so on. This method involves a global search, which involves locating outliers within the moving window, setting constraints on parameter values, and detecting and repairing triple-frequency cycle slips.

### Cycle slip detection and repair process

**Figure 2:**
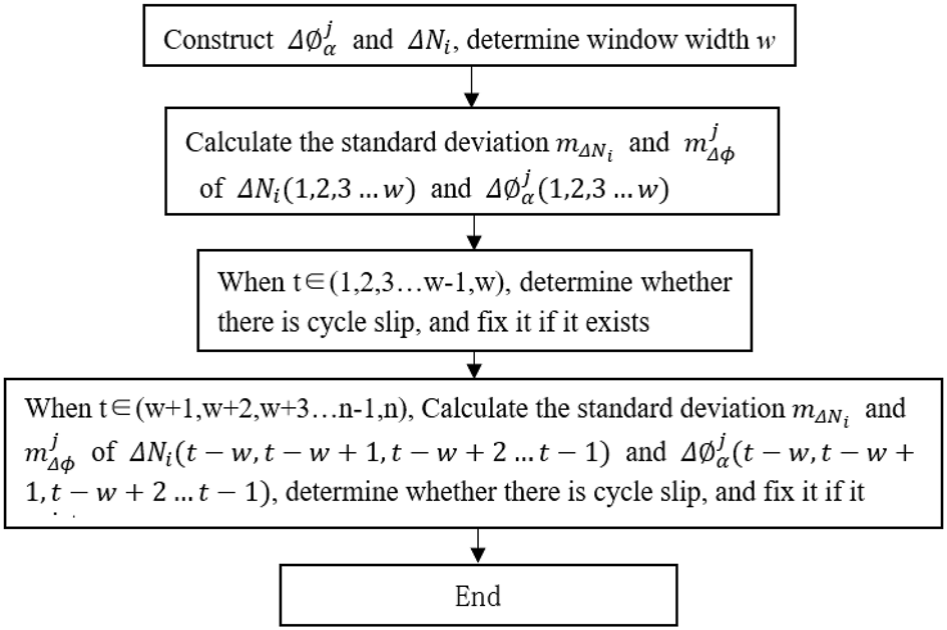
Algorithm flowchart.

The algorithm flowchart is shown in Fig. 2, and the specific steps are as follows:

1. Based on the selected coefficients $\mathrm{\alpha }$, construct ${\Delta \varnothing }_{\alpha }^{j}$ and ${\Delta N}_{i}$ for GPS and BDS, and determine the moving window width $w$, define the XZQZ[a, b] function (rounded towards the middle of two numbers a and b).
2. Calculate the standard deviations ${m}_{{\Delta N}_{i}}$ and ${m}_{\Delta \phi }^{j}$ of ${\Delta N}_{i}({1,2},3\ldots \ldots w)$ and ${\Delta \varnothing }_{\alpha }^{j}(\mathrm{1,2},3\ldots \ldots w)$.
3. For any epoch $t\in (\mathrm{1,2},3\ldots \ldots w-1,w)$, if any of the three combinations $\left|\Delta {\varnothing }_{\alpha }^{j}(t)\right|>3{m}_{\Delta \phi }^{j}$, it indicates that there is a suspected cycle slip at epoch $t$. The integer solution satisfying Eqs. (11) and (13) below is the cycle slip. Detect and calculate $t$ epoch ${\Delta N}_{i}$ according to Eqs. (11) and (13); Simultaneously repair ${\Delta N}_{i}(t)$ and $\Delta {\varnothing }_{\alpha }^{j}(t)$ in the original sequence according to Eqs. (12) and (14); recalculate the standard deviations ${m}_{{\Delta N}_{i}}$ and ${m}_{\Delta \phi }^{j}$ of ${\Delta N}_{i}(\mathrm{1,2},3\ldots w)$ and ${\Delta \varnothing }_{\alpha }^{j}(\mathrm{1,2},3\ldots w)$ until $t=w$.
  11 $$\left\{\begin{array}{c}\begin{array}{c}{\Delta N}_{1}\in XZQZ\left[{\Delta N}_{1}(t)-3{m}_{{\Delta N}_{1}} {\Delta N}_{1}(t)+3{m}_{{\Delta N}_{1}}\right]\\ {\Delta N}_{2}\in XZQZ\left[{\Delta N}_{2}(t)-3{m}_{{\Delta N}_{2}} {\Delta N}_{2}(t)+3{m}_{{\Delta N}_{2}}\right]\end{array}\\ \begin{array}{c}{\Delta N}_{3}\in XZQZ\left[{\Delta N}_{3}(t)-3{m}_{{\Delta N}_{3}} {\Delta N}_{3}(t)+3{m}_{{\Delta N}_{3}}\right]\\ {\left[\begin{array}{c}-3{m}_{\Delta \phi }^{1}\\ -3{m}_{\Delta \phi }^{2}\\ -3{m}_{\Delta \phi }^{3}\end{array}\right]}_{GPS}<{\left[\begin{array}{c}\Delta {\varnothing }_{\alpha }^{1}(t)\\ \Delta {\varnothing }_{\alpha }^{2}(t)\\ \Delta {\varnothing }_{\alpha }^{3}(t)\end{array}\right]}_{GPS}-\left[\begin{array}{ccc}{\lambda }_{1}& -6{\lambda }_{2}& 5{\lambda }_{3}\\ {\lambda }_{1}& -5{\lambda }_{2}& 4{\lambda }_{3}\\ {\lambda }_{1}& -4{\lambda }_{2}& 3{\lambda }_{3}\end{array}\right]{\left[\begin{array}{c}\Delta {N}_{1}\\ \Delta {N}_{2}\\ {\Delta N}_{3}\end{array}\right]}_{GPS}<{\left[\begin{array}{c}3{m}_{\Delta \phi }^{1}\\ 3{m}_{\Delta \phi }^{2}\\ 3{m}_{\Delta \phi }^{3}\end{array}\right]}_{GPS}\end{array}\end{array}\right.$$
  12 $$\left\{\begin{array}{c}\begin{array}{c}{\Delta N}_{1}(t)={\Delta N}_{1}(t)-{\Delta N}_{1}\\ {\Delta N}_{2}(t)={\Delta N}_{2}(t)-{\Delta N}_{2}\end{array}\\ \begin{array}{c}{\Delta N}_{3}(t)={\Delta N}_{3}(t)-{\Delta N}_{3}\\ {\left[\begin{array}{c}\Delta {\varnothing }_{\alpha }^{1}(t)\\ \Delta {\varnothing }_{\alpha }^{2}(t)\\ \Delta {\varnothing }_{\alpha }^{3}(t)\end{array}\right]}_{GPS}={\left[\begin{array}{c}\Delta {\varnothing }_{\alpha }^{1}(t)\\ \Delta {\varnothing }_{\alpha }^{2}(t)\\ \Delta {\varnothing }_{\alpha }^{3}(t)\end{array}\right]}_{GPS}-\left[\begin{array}{ccc}{\lambda }_{1}& -6{\lambda }_{2}& 5{\lambda }_{3}\\ {\lambda }_{1}& -5{\lambda }_{2}& 4{\lambda }_{3}\\ {\lambda }_{1}& -4{\lambda }_{2}& 3{\lambda }_{3}\end{array}\right]{\left[\begin{array}{c}\Delta {N}_{1}\\ \Delta {N}_{2}\\ {\Delta N}_{3}\end{array}\right]}_{GPS}\end{array}\end{array}\right.$$
  13 $$\left\{\begin{array}{c}\begin{array}{c}{\Delta N}_{1}\in XZQZ\left[{\Delta N}_{1}(t)-3{m}_{{\Delta N}_{1}} {\Delta N}_{1}(t)+3{m}_{{\Delta N}_{1}}\right]\\ {\Delta N}_{2}\in XZQZ\left[{\Delta N}_{2}(t)-3{m}_{{\Delta N}_{2}} {\Delta N}_{2}(t)+3{m}_{{\Delta N}_{2}}\right]\end{array}\\ \begin{array}{c}{\Delta N}_{3}\in XZQZ\left[{\Delta N}_{3}(t)-3{m}_{{\Delta N}_{3}} {\Delta N}_{3}(t)+3{m}_{{\Delta N}_{3}}\right]\\ {\left[\begin{array}{c}-3{m}_{\Delta \phi }^{1}\\ -3{m}_{\Delta \phi }^{2}\\ -3{m}_{\Delta \phi }^{3}\end{array}\right]}_{BDS}<{\left[\begin{array}{c}\Delta {\varnothing }_{\alpha }^{1}(t)\\ \Delta {\varnothing }_{\alpha }^{2}(t)\\ \Delta {\varnothing }_{\alpha }^{3}(t)\end{array}\right]}_{BDS}-\left[\begin{array}{ccc}{\lambda }_{1}& 2{\lambda }_{2}& -3{\lambda }_{3}\\ {\lambda }_{1}& 3{\lambda }_{2}& -4{\lambda }_{3}\\ {\lambda }_{1}& 4{\lambda }_{2}& -5{\lambda }_{3}\end{array}\right]{\left[\begin{array}{c}\Delta {N}_{1}\\ \Delta {N}_{2}\\ {\Delta N}_{3}\end{array}\right]}_{BDS}<{\left[\begin{array}{c}3{m}_{\Delta \phi }^{1}\\ 3{m}_{\Delta \phi }^{2}\\ 3{m}_{\Delta \phi }^{3}\end{array}\right]}_{BDS}\end{array}\end{array}\right.$$
  14 $$\left\{\begin{array}{c}\begin{array}{c}{\Delta N}_{1}(t)={\Delta N}_{1}(t)-{\Delta N}_{1}\\ {\Delta N}_{2}(t)={\Delta N}_{2}(t)-{\Delta N}_{2}\end{array}\\ \begin{array}{c}{\Delta N}_{3}(t)={\Delta N}_{3}(t)-{\Delta N}_{3}\\ {\left[\begin{array}{c}\Delta {\varnothing }_{\alpha }^{1}(t)\\ \Delta {\varnothing }_{\alpha }^{2}(t)\\ \Delta {\varnothing }_{\alpha }^{3}(t)\end{array}\right]}_{BDS}={\left[\begin{array}{c}\Delta {\varnothing }_{\alpha }^{1}(t)\\ \Delta {\varnothing }_{\alpha }^{2}(t)\\ \Delta {\varnothing }_{\alpha }^{3}(t)\end{array}\right]}_{BDS}-\left[\begin{array}{ccc}{\lambda }_{1}& 2{\lambda }_{2}& -3{\lambda }_{3}\\ {\lambda }_{1}& 3{\lambda }_{2}& -4{\lambda }_{3}\\ {\lambda }_{1}& 4{\lambda }_{2}& -5{\lambda }_{3}\end{array}\right]{\left[\begin{array}{c}\Delta {N}_{1}\\ \Delta {N}_{2}\\ {\Delta N}_{3}\end{array}\right]}_{BDS}\end{array}\end{array}\right.$$
4. For any epoch $t\in (w+1,w+2,w+3\ldots n)$, calculate the standard deviations ${m}_{{\Delta N}_{i}}$ and ${m}_{\Delta \phi }^{j}$ of ${\Delta N}_{i}(t-w,t-w+1,t-w+2\ldots t-1)$ and ${\Delta \varnothing }_{\alpha }^{j}(t-w,t-w+1,t-w+2\ldots t-1)$, if any of the three combinations $\left|\Delta {\varnothing }_{\alpha }^{j}(t)\right|>3{m}_{\Delta \phi }^{j}$, it indicates that there is a suspected cycle slip at epoch $t$. Detect and repair cycle slip according to Eqs. (11) to (14) until $t=n$.

### Data testing and analysis

#### Data source and experimental description

To validate the GPS/BDS triple-frequency cycle slip detection and repair method described in this article, we utilized data from two Hong Kong CORS stations HKKT and HKMW, which were observed on the 1st and 54th days of 2023, respectively. The HKKT station was observed for 5 h from 0:00 to 5:00, while the HKMW station was observed for 8 h from 5:00 to 13:00 with a sampling rate of 1 Hz. Table 3 displays the essential information of the satellites used in the experiment, and the data can be downloaded from ftp://ftp.geodetic.gov.hk/. The moving window width was set to w = 30 epochs, which is equivalent to 30 s. The experiment was divided into five groups, as shown in Table 4. During data processing, P-code was used to replace pseudo-range observations in GPS, and the average of triple-frequency pseudo-range was used to replace pseudo-range observations in BDS.

**Table 3:** Basic information of experimental satellites.

System	$PRN$	Observation value type	Remarks
GPS	G10	C1 L1 P2 L2 C5 L5	Block IIF
BDS	C04	C2I C6I C7I L2I L6I L7I	GEO
	C13 C16		IGSO
	C12		MEO

**Table 4:** Basic information of experimental design.

Experiment number	$PRN$	Add cycle slip	Number of cycle slip
1	G10 C04 C12 C16	Within 10 cycles, random epoch random size	100
2	G10 C04 C12 C16	4–10 cycles, random epoch random size	100
3	G10 C04 C12 C16	2–4 cycles, random epoch random size	100
4	G10 C04 C12 C16	Within 2 cycles, random epoch random size	100
5	C04 C12 C13	Fixed epoch fixed size within 10 cycles	100

#### Experiment 1

**Figure 3:**
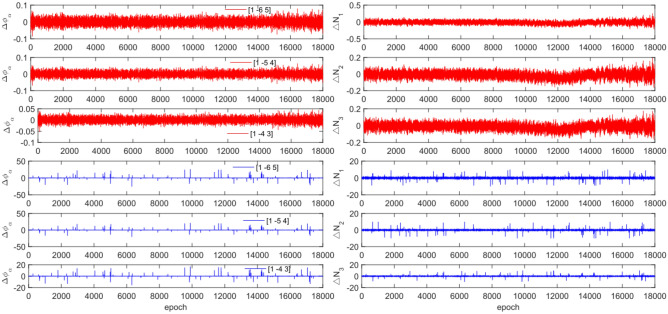
Comparison of ${\Delta \mathrm{\varnothing }}_{\alpha }^{j}$ and ${\Delta N}_{i}$ before and after adding cycle slips to G10 (the first column represents ${\Delta \mathrm{\varnothing }}_{\alpha }^{j}$ and the second column represents ${\Delta N}_{i}$; The first three rows and the last three rows represent before and after adding the cycle slips respectively).

**Figure 4:**
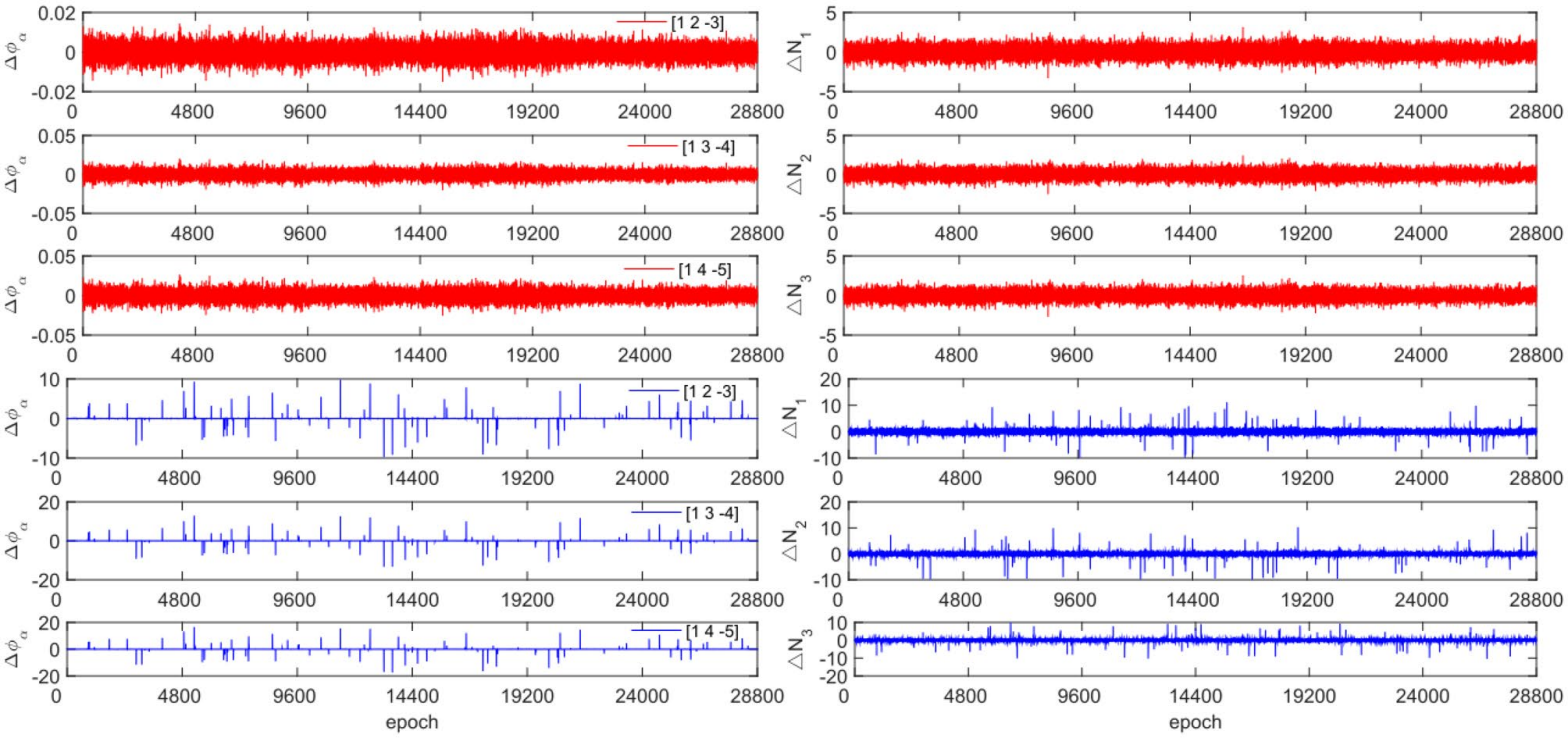
Comparison of ${\Delta \mathrm{\varnothing }}_{\alpha }^{j}$ and ${\Delta N}_{i}$ before and after adding cycle slips to C04 (the first column represents ${\Delta \mathrm{\varnothing }}_{\alpha }^{j}$ and the second column represents ${\Delta N}_{i}$; The first three rows and the last three rows represent before and after adding the cycle slips respectively).

**Figure 5:**
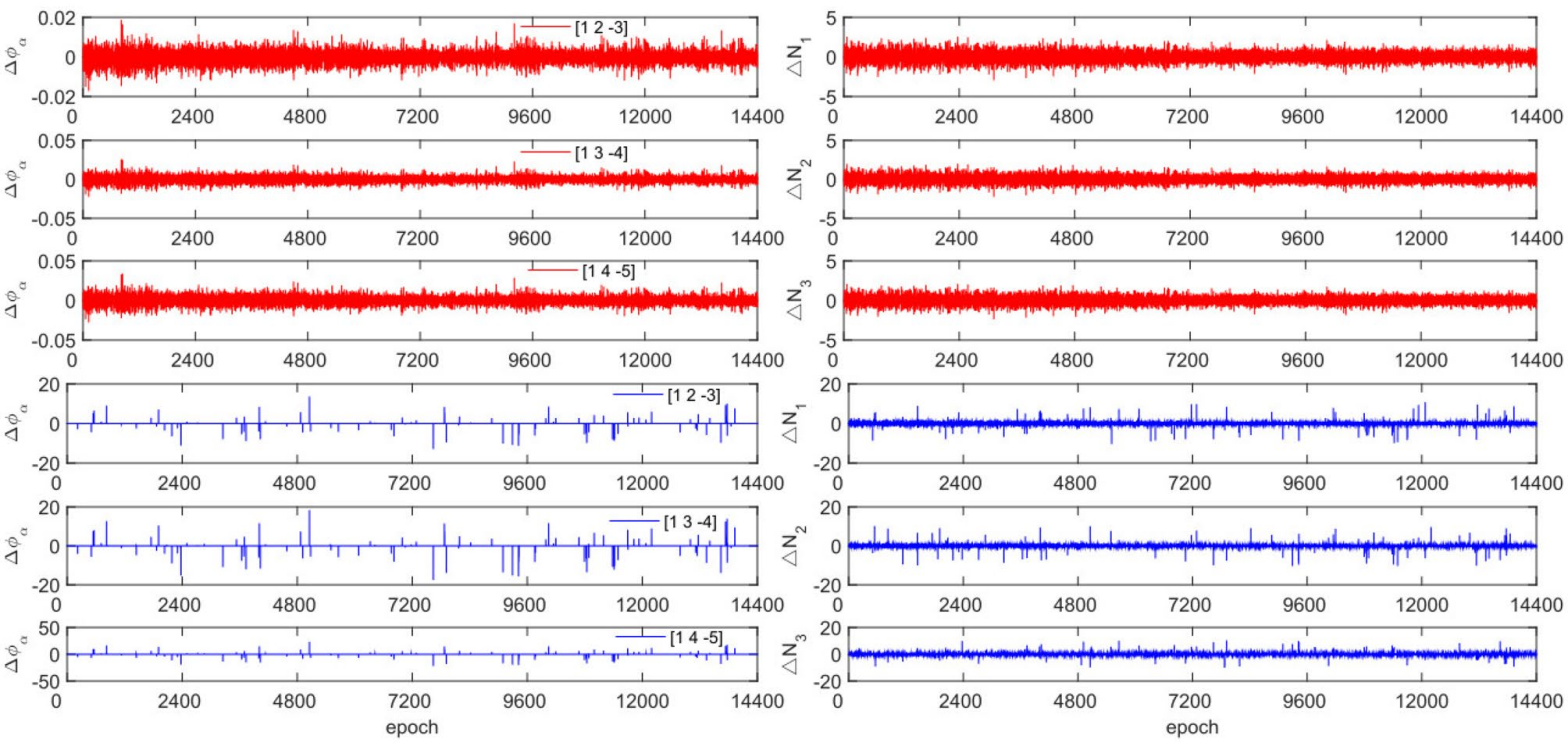
Comparison of ${\Delta \mathrm{\varnothing }}_{\alpha }^{j}$ and ${\Delta N}_{i}$ before and after adding cycle slips to C12 (the first column represents ${\Delta \mathrm{\varnothing }}_{\alpha }^{j}$ and the second column represents ${\Delta N}_{i}$; The first three rows and the last three rows represent before and after adding the cycle slips respectively).

**Figure 6:**
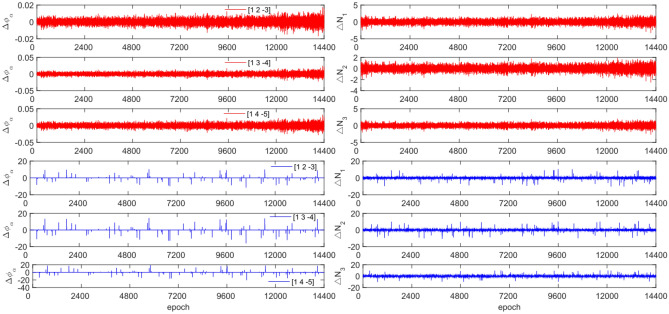
Comparison of ${\Delta \mathrm{\varnothing }}_{\alpha }^{j}$ and ${\Delta N}_{i}$ before and after adding cycle slips to C16 (the first column represents ${\Delta \mathrm{\varnothing }}_{\alpha }^{j}$ and the second column represents ${\Delta N}_{i}$; the first three rows and the last three rows represent before and after adding the cycle slips respectively).

The GPS and BDS data utilize different combination coefficients. Specifically, GPS uses [1 −6 5] [1 −5 4], and [1 −4 3], while BDS uses [1 2 −3], [1 3 −4], and [1 4 −3]. Furthermore, 100 sets of random cycle slips were artificially added within the range of [−10, 10]. Figures 3, 4, 5 and 6 compare the ${\Delta \varnothing }_{\alpha }^{j}$ and ${\Delta N}_{i}$ before and after cycle slips were added to the G10, C04, C12, and C16. The cycle slip detection method is compared with the direct rounding method in Table 5.

**Table 5:** Detection effect and comparison of experiment 1.

PRN	Total cycle slips	Moving window global search method					Direct rounding method
		Successful	False detection	Non-detection	Multiple detection	Success rate (%)	Success rate (%)
G10	100	99	0	1	0	99.0	99.0
C04		100	0	0	0	100.0	97.0
C12		94	1	5	0	94.0	93.0
C16		98	1	1	0	98.0	97.0
Average						97.8	96.5

False detection indicates that both the detection position and size are incorrect. Non-detection indicates the presence of a cycle slip without detecting any cycle slip, while multiple detections indicate an error in detecting cycle slip beyond the cycle slip position.

Figures 3, 4, 5 and 6 demonstrate that cycle slips have a significant impact on the amplitude of ${\Delta \mathrm{\varnothing }}_{\alpha }^{j}$ and ${\Delta N}_{i}$. The experiment achieved a detection success rate of over 94.0%, but there were some cases of false and non-detections. The analysis shows that the BDS system has more false detections than GPS due to the use of pseudo-range for cycle slip estimation calculation, while GPS uses P-code. Furthermore, it is important to note that small cycle slips may be obscured by observation noise. The experimental results show that the average success rate is 1.3% higher than the direct rounding method used.

#### Experiment 2

**Figure 7:**
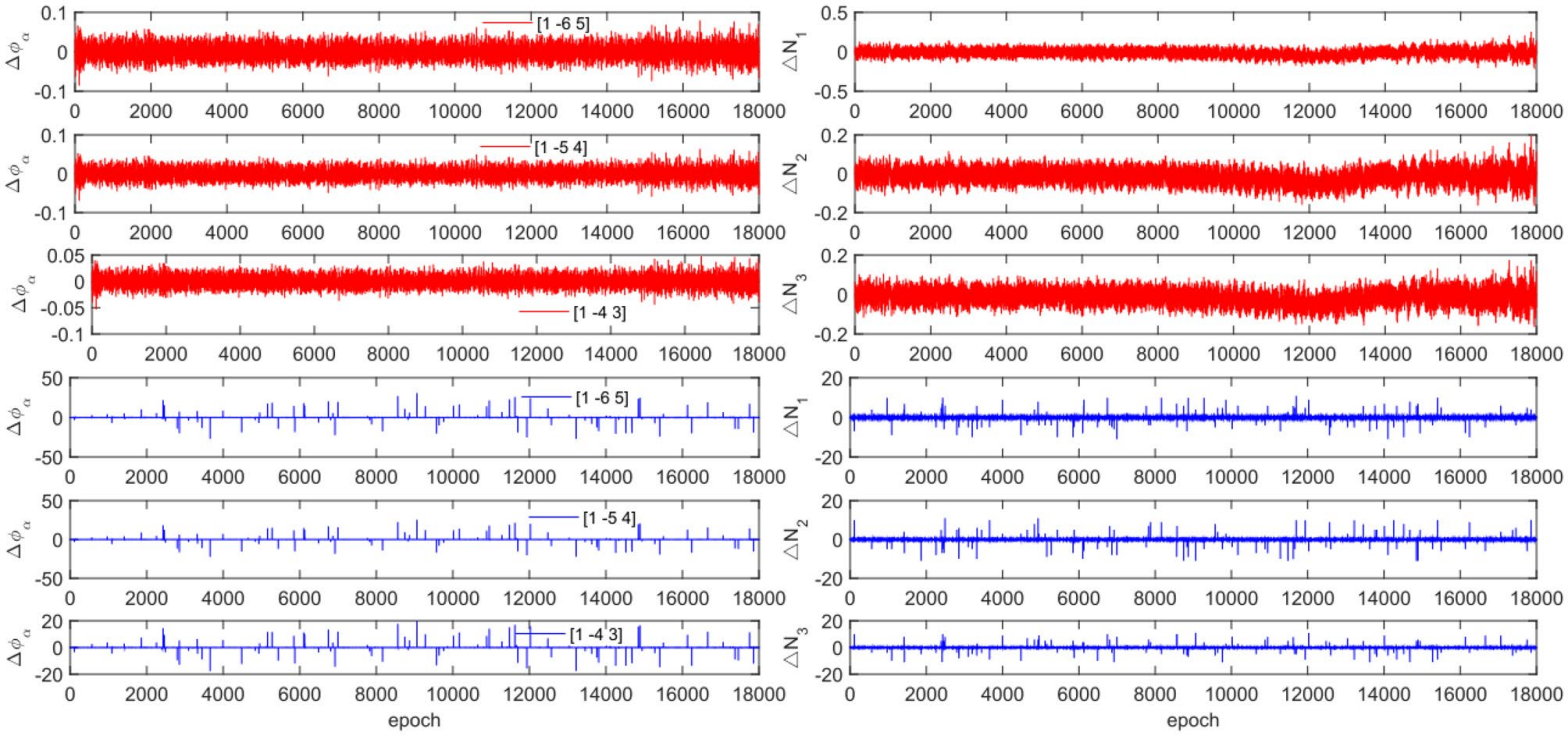
Comparison of ${\Delta \mathrm{\varnothing }}_{\alpha }^{j}$ and ${\Delta N}_{i}$ before and after adding cycle slips to G10 (the first column represents ${\Delta \mathrm{\varnothing }}_{\alpha }^{j}$ and the second column represents ${\Delta N}_{i}$; the first three rows and the last three rows represent before and after adding the cycle slips respectively).

**Figure 8:**
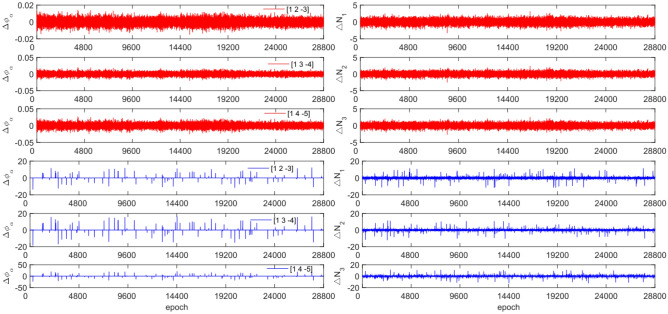
Comparison of ${\Delta \mathrm{\varnothing }}_{\alpha }^{j}$ and ${\Delta N}_{i}$ before and after adding cycle slips to C04 (the first column represents ${\Delta \mathrm{\varnothing }}_{\alpha }^{j}$ and the second column represents ${\Delta N}_{i}$; the first three rows and the last three rows represent before and after adding the cycle slips respectively).

**Figure 9:**
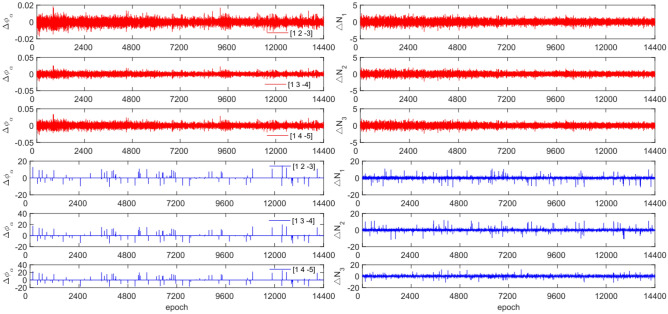
Comparison of ${\Delta \mathrm{\varnothing }}_{\alpha }^{j}$ and ${\Delta N}_{i}$ before and after adding cycle slips to C10 (the first column represents ${\Delta \mathrm{\varnothing }}_{\alpha }^{j}$ and the second column represents ${\Delta N}_{i}$; the first three rows and the last three rows represent before and after adding the cycle slips respectively).

**Figure 10:**
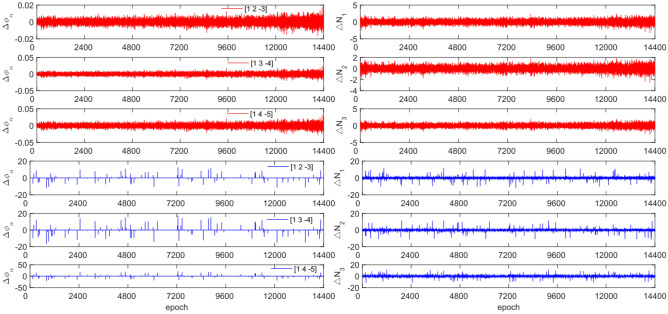
Comparison of ${\Delta \mathrm{\varnothing }}_{\alpha }^{j}$ and ${\Delta N}_{i}$ before and after adding cycle slips to C16 (the first column represents ${\Delta \mathrm{\varnothing }}_{\alpha }^{j}$ and the second column represents ${\Delta N}_{i}$; the first three rows and the last three rows represent before and after adding the cycle slips respectively).

The experiment used the same original observation data and combination coefficients as Experiment 1 for each satellite, but with added cycle slip sizes of [−10 −4] and [4 10] cycles. Figures 7, 8, 9 and 10 compared the G10, C04, C12 and C16 before and after adding cycle slips. Table 6 shows the detection effect of this experiment.

**Table 6:** Detection effect and comparison of experiment 2.

PRN	Total cycle slips	Moving window global search method					Direct rounding method
		Successful	False detection	Non-detection	Multiple detection	Success rate (%)	Success rate (%)
G10	100	100	0	0	0	100.0	98.0
C04		99	0	1	2	99.0	98.0
C12		99	0	1	0	99.0	98.0
C16		97	3	0	0	97.0	95.0
Average						98.8	97.2

From Figs. 7, 8, 9 and 10, it can be seen that before and after adding cycle slips, the amplitudes of ${\Delta \mathrm{\varnothing }}_{\alpha }^{j}$ and ${\Delta N}_{i}$ significantly increase. However, GPS has a higher success rate than BDS in terms of detection effectiveness, with BDS experiencing false detections, non-detections, and multiple detections. Despite this, the overall detection success rate is over 97.0%. The experimental results demonstrate that this method has an average success rate 1.6% higher than the direct rounding method.

#### Experiment 3

**Figure 11:**
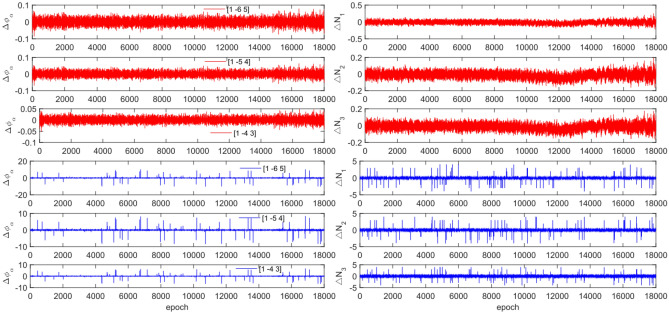
Comparison of ${\Delta \mathrm{\varnothing }}_{\alpha }^{j}$ and ${\Delta N}_{i}$ before and after adding cycle slips to G10 (the first column represents ${\Delta \mathrm{\varnothing }}_{\alpha }^{j}$ and the second column represents ${\Delta N}_{i}$; the first three rows and the last three rows represent before and after adding the cycle slips respectively).

**Figure 12:**
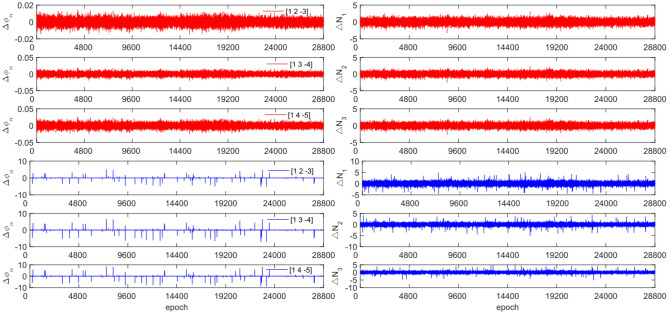
Comparison of ${\Delta \mathrm{\varnothing }}_{\alpha }^{j}$ and ${\Delta N}_{i}$ before and after adding cycle slips to C04 (the first column represents ${\Delta \mathrm{\varnothing }}_{\alpha }^{j}$ and the second column represents ${\Delta N}_{i}$; the first three rows and the last three rows represent before and after adding the cycle slips respectively).

**Figure 13:**
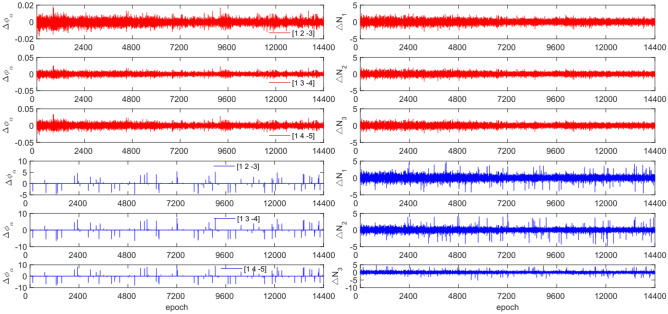
Comparison of ${\Delta \mathrm{\varnothing }}_{\alpha }^{j}$ and ${\Delta N}_{i}$ before and after adding cycle slips to C12 (the first column represents ${\Delta \mathrm{\varnothing }}_{\alpha }^{j}$ and the second column represents ${\Delta N}_{i}$; the first three rows and the last three rows represent before and after adding the cycle slips respectively).

**Figure 14:**
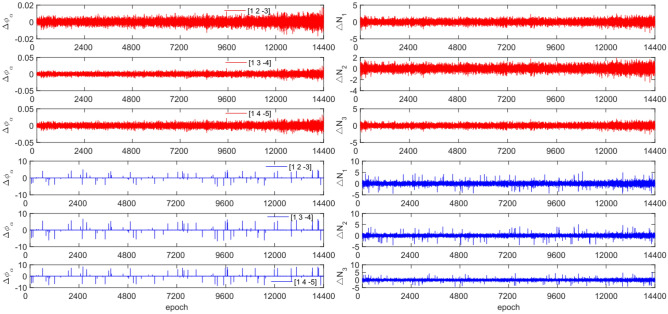
Comparison of ${\Delta \mathrm{\varnothing }}_{\alpha }^{j}$ and ${\Delta N}_{i}$ before and after adding cycle slips to C16 (the first column represents ${\Delta \mathrm{\varnothing }}_{\alpha }^{j}$ and the second column represents ${\Delta N}_{i}$; the first three rows and the last three rows represent before and after adding the cycle slips respectively).

The experiment used the same original observations and combination coefficients for each satellite as in Experiment 1, but with the addition of cycle slip sizes of [−4 −2] and [2 4] cycles. Figures 11, 12, 13 and 14 compare the G10, C04, C12 and C16 satellites before and after adding the cycle slips. The detection results are presented in Table 7.

**Table 7:** Detection effect and comparison of experiment 3.

PRN	Total cycle slips	Moving window global search method					Direct rounding method
		Successful	False detection	Non-detection	Multiple detection	Success rate (%)	Success rate (%)
G10	100	99	0	1	0	99.0	99.0
C04		97	0	3	0	97.0	95.0
C12		98	1	1	1	98.0	98.0
C16		96	1	2	0	96.0	95.0
Average						97.5	96.8

Figures 11, 12, 13 and 14 show that the addition of cycle slips significantly affects the change in ${\Delta \varnothing }_{\alpha }^{j}$ with a relatively large amplitude, ${\Delta N}_{i}$ shows a slight increase in amplitude. Undetected situations were observed for multiple system satellites in terms of detection effectiveness. It is analysed that the added cycle slip size is submerged by noise, resulting in false and multiple detections by BDS. The accuracy of the pseudo-range is significantly lower than that of the P-code. However, the overall success rate exceeds 96.0%. The experimental results demonstrate that this method has an average success rate 0.7% higher than that of the direct rounding method.

#### Experiment 4

**Figure 15:**
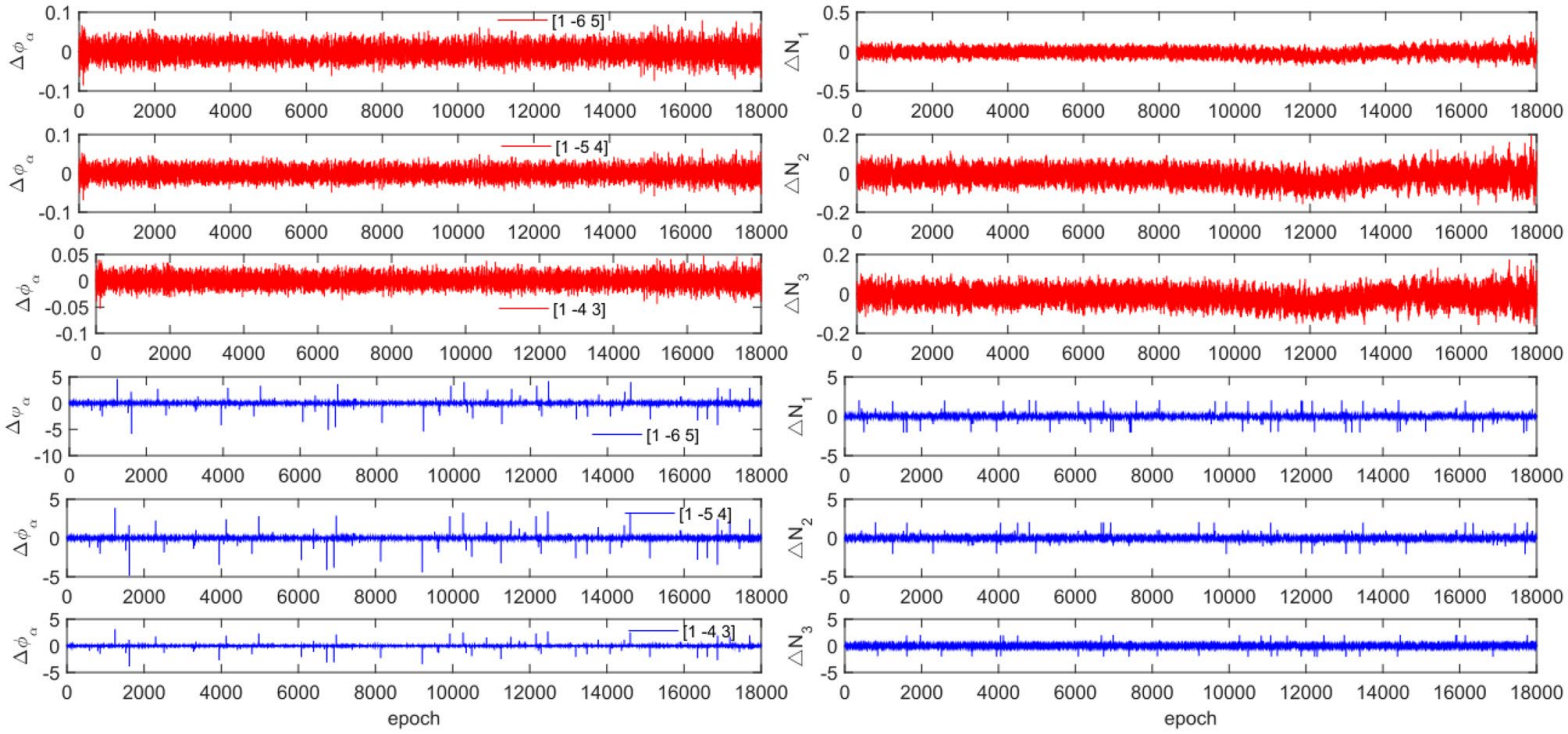
Comparison of ${\Delta \mathrm{\varnothing }}_{\alpha }^{j}$ and ${\Delta N}_{i}$ before and after adding cycle slips to G10 (the first column represents ${\Delta \mathrm{\varnothing }}_{\alpha }^{j}$ and the second column represents ${\Delta N}_{i}$; the first three rows and the last three rows represent before and after adding the cycle slips respectively).

**Figure 16:**
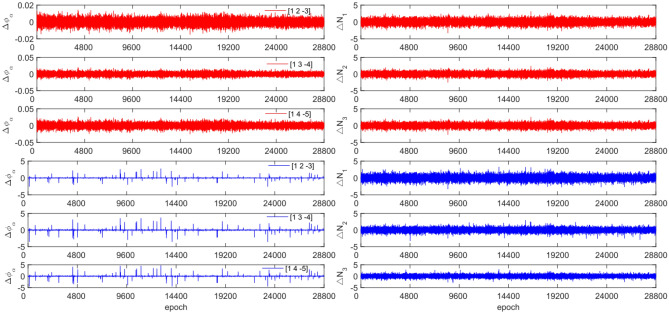
Comparison of ${\Delta \mathrm{\varnothing }}_{\alpha }^{j}$ and ${\Delta N}_{i}$ before and after adding cycle slips to C04 (the first column represents ${\Delta \mathrm{\varnothing }}_{\alpha }^{j}$ and the second column represents ${\Delta N}_{i}$; the first three rows and the last three rows represent before and after adding the cycle slips respectively).

**Figure 17:**
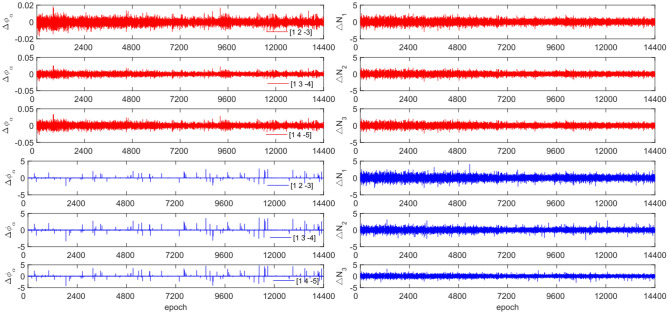
Comparison of ${\Delta \mathrm{\varnothing }}_{\alpha }^{j}$ and ${\Delta N}_{i}$ before and after adding cycle slips to C12 (the first column represents ${\Delta \mathrm{\varnothing }}_{\alpha }^{j}$ and the second column represents ${\Delta N}_{i}$; the first three rows and the last three rows represent before and after adding the cycle slips respectively).

**Figure 18:**
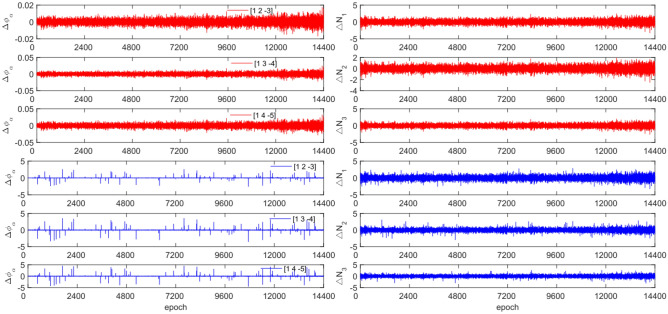
Comparison of ${\Delta \mathrm{\varnothing }}_{\alpha }^{j}$ and ${\Delta N}_{i}$ before and after adding cycle slips to C16 (the first column represents ${\Delta \mathrm{\varnothing }}_{\alpha }^{j}$ and the second column represents ${\Delta N}_{i}$; the first three rows and the last three rows represent before and after adding the cycle slips respectively).

To test the effectiveness of the method described in this article in detecting and repairing small cycle slips, cycle slip sizes of [−2 2] cycles were added in the experimental design. The observation data and combination coefficients used for each satellite in this experiment are identical to those used in Experiment 1. Figures 15, 16, 17 and 18 compare the G10, C04, C12, and C16 satellites before and after adding the cycle slips. The detection effect of this experiment is shown in Table 8.

**Table 8:** Detection effect and comparison in experiment 4.

PRN	Total cycle slips	Moving window global search method					Direct rounding method
		Successful	False detection	Non-detection	Multiple detection	Success rate (%)	Success rate (%)
G10	100	99	0	1	0	99.0	98.0
C04		94	0	6	0	94.0	92.0
C12		92	0	8	0	92.0	88.0
C16		95	0	5	0	95.0	92.0
Average						95.0	92.5

From Figs. 15, 16, 17 and 18, it can be seen that before and after adding cycle slips, ${\Delta \varnothing }_{\alpha }^{j}$ changes significantly and the curve has sharp parts, the change in ${\Delta N}_{i}$ is gentle, with almost the same shape before and after. From the perspective of detection effectiveness, the method of using P-code to constrain GPS cycle estimation for the added small cycle slips is significantly higher than that of BDS using pseudo-range average constraint. In terms of the number of undetected cycles, BDS satellites are significantly higher than GPS satellites. The overall success rate is over 92.0%. From the experimental results, it can be seen that the average success rate of this method is 2.5% higher than that of the direct rounding method.

#### Experiment 5

**Figure 19:**
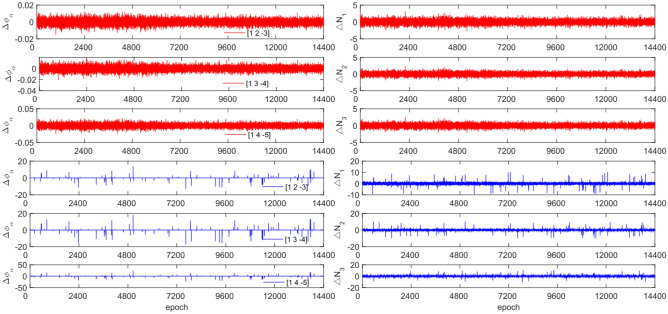
Comparison of ${\Delta \mathrm{\varnothing }}_{\alpha }^{j}$ and ${\Delta N}_{i}$ before and after adding cycle slips to C04 (the first column represents ${\Delta \mathrm{\varnothing }}_{\alpha }^{j}$ and the second column represents ${\Delta N}_{i}$; the first three rows and the last three rows represent before and after adding the cycle slips respectively).

**Figure 20:**
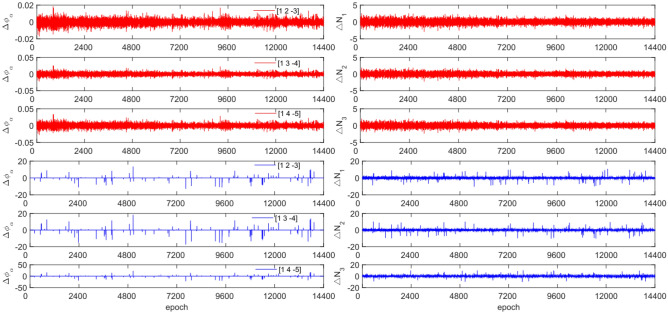
Comparison of ${\Delta \mathrm{\varnothing }}_{\alpha }^{j}$ and ${\Delta N}_{i}$ before and after adding cycle slips to C12 (the first column represents ${\Delta \mathrm{\varnothing }}_{\alpha }^{j}$ and the second column represents ${\Delta N}_{i}$; the first three rows and the last three rows represent before and after adding the cycle slips respectively).

**Figure 21:**
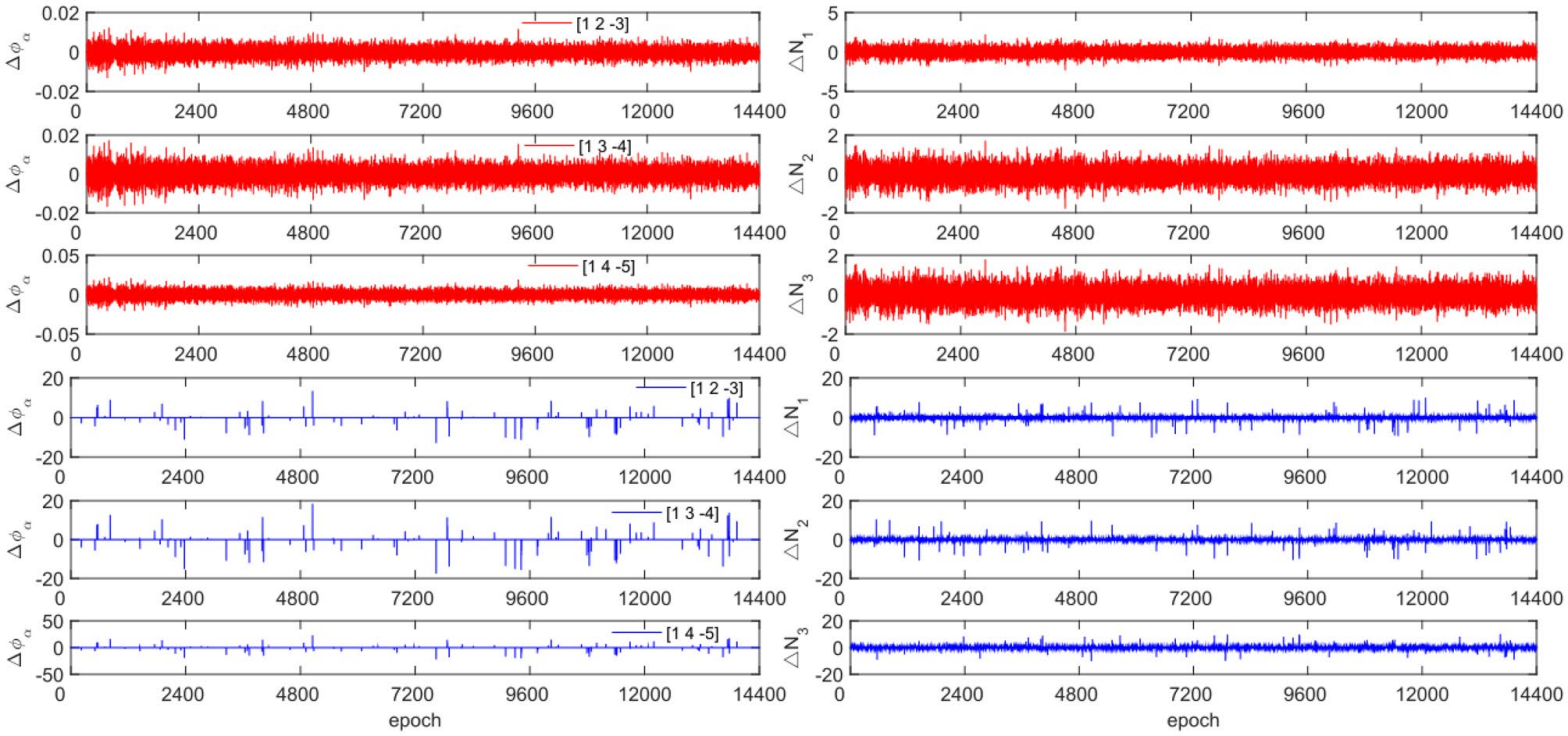
Comparison of ${\Delta \mathrm{\varnothing }}_{\alpha }^{j}$ and ${\Delta N}_{i}$ before and after adding cycle slips to C13 (the first column represents ${\Delta \mathrm{\varnothing }}_{\alpha }^{j}$ and the second column represents ${\Delta N}_{i}$; the first three rows and the last three rows represent before and after adding the cycle slips respectively).

To assess the detection and repair effects of three types of satellite cycle slips in the BDS system, this experiment analysed 4 h (09:00–13:00) of observation data from C04, C12 and C13. For comparative analysis, 100 sets of fixed epoch fixed cycle slips within the range of [-10, 10] were added. Figures 19, 20 and 21 compare ${\Delta \mathrm{\varnothing }}_{\alpha }^{j}$ and ${\Delta N}_{i}$ before and after the addition of cycle slip of C04, C12 and C13. The detection effect of this experiment is shown in Table 9.

**Table 9:** Detection effect and comparison of experiment 5.

PRN	Total cycle slips	Moving window global search method					Direct rounding method
		Successful	False detection	Non-detection	Multiple detection	Success rate (%)	Success rate (%)
C04	100	99	0	1	0	99.0	98.0
C12		95	0	5	0	95.0	94.0
C13		97	0	3	0	97.0	93.0
Average						97.0	95.0

Figures 19, 20 and 21 show that the same size cycle slip method with fixed epochs did not result in any false or multiple detections. However, there were still some cases of undetected detections. Experiment 5 demonstrates that the detection success rate of this method is consistent across different types of BDS satellites, with a success rate of over 95.0%. The experimental results demonstrate that this method has an average success rate 2.0% higher than the direct rounding method.

#### Analysis

(1) The results of Experiments 1 to 5 demonstrate that the moving window global search method is effective in detecting and repairing cycle slips within 10 cycles, 4–10 cycles, 2–4 cycles, and within 2 cycles, with average detection success rates of 97.2%, 98.8%, 97.5%, 95.0% and 97.0%, respectively. No issues with multiple values were observed. (2) The average detection success rate across all five experiments was 97.1%, which is 1.5% higher than the success rate of the direct rounding method at 95.6%. (3) The success rate of GPS cycle slip detection using P-code constraints is higher than that of BDS cycle slip detection using pseudo range constraints, with an average increase of about 1.8%. (4) Experiments 1 to 4 showed that using P-code instead of pseudo-range for GPS cycle slip detection resulted in an average success rate of 99.2%, which is 0.7% higher than the direct rounding method of 98.5%. The detection effect is equivalent. Additionally, using the average of triple-frequency pseudo-range instead of BDS for cycle slip detection resulted in an average success rate of 96.6%, which is 1.8% higher than the direct rounding method of 94.8%. (5) In general, the moving window global search method has a higher success rate than the direct rounding method.

## Conclusion

Based on the moving window global search method, the following conclusions were drawn from the experimental analysis:

1. The GPS and BDS systems both utilize different combination coefficients to form the GF model can effectively reduce the impact of ionospheric interference. By implementing a moving window and constraining the search range using the repaired sequence standard deviation, cycle slips can be accurately detected and repaired. The success rate range from 92.0% to 100.0%, demonstrating its feasibility and effectiveness.
2. Applying the "3σ" criterion to constrain the search range in a moving window can effectively avoid the multi value problem in cycle slip detection process.
3. The moving window global search method is able to detect and repair both small cycle slips within 2 cycles and larger cycle slips exceeding 2 cycles.

The detection and repair of cycle slips in BDS with more than triple-frequency, dynamic modes, and different sampling conditions will be our future focus of research.

## Data Availability

All data and materials can be consulted with the corresponding author. The original data can be downloaded publicly, the download website is: ftp://ftp.geodetic.gov.hk/. Other materials can be obtained by contacting the corresponding author D.H. (email: dwhuang81@163.com).
